# Individual Ultrasonographic Characteristics of Thyroid Nodules and Their Cytopathological Correlation to Determine Malignancy Risk

**DOI:** 10.7759/cureus.63918

**Published:** 2024-07-05

**Authors:** Miguel Ángel Castilla Villanueva, Dania Guadalupe Solis Cano, Ana Amador Martínez, Marco Antonio Téliz Meneses, Jesús Baquera-Heredia, Cesar Eduardo Vallin Orozco, Mónica Loya Ceballos

**Affiliations:** 1 Radiology, Monterrey Institute of Technology and Higher Education, Mexico City, MEX; 2 Radiology, The American British Cowdray Medical Center, Mexico City, MEX; 3 Pathology, The American British Cowdray Medical Center, Mexico City, MEX

**Keywords:** the bethesda system for reporting thyroid cytopathology (tbsrtc), thyroid nodule, acr tirads, thyroid cancer, ultrasonography, thyroid cytology, fine needle aspiration biopsy (fnac), bethesda, american college of radiology

## Abstract

Background

Ultrasonographic evaluation of thyroid nodules is challenging due to their high frequency and low malignancy rate. The risk stratification system developed by the American College of Radiology (ACR) Thyroid Imaging Reporting and Data System (TI-RADS) focuses on addressing the primary contemporary objectives for these lesions, aiming to decrease unnecessary biopsies while maintaining a similar specificity compared with other risk stratification systems. Generally, when indicative of malignancy by ultrasound findings, the next best step in management is an evaluation by fine needle aspiration biopsy (FNAB) and cytological analysis with The Bethesda System for Reporting Thyroid Cytopathology (TBSRTC) results that determine further evaluation requirements, actions that are based on the risk of malignancy (ROM) of the assigned category, which could include surgical intervention.

Objectives

To validate and analyze the individual impact of each ultrasonographic finding indicative of malignancy in the ACR TI-RADS guidelines based on their respective correlation with results obtained by TBSRTC.

Materials and method

Reports for 212 thyroid ultrasound-guided FNABs from 2018 to 2020 were assessed. Only 117 had both ACR TI-RADS and TBSRTC reports available and were analyzed. Nodules were divided into two groups: ROM < 5% (Bethesda 1, 2; n = 58), and ROM > 5% (Bethesda 3, 4, 5, 6; n = 59). Statistical analysis was performed using the x^2^ test and bivariate logistic regression model for each characteristic included in ACR TI-RADS.

Results

Individual ultrasound characteristics with a more pronounced distribution towards the Bethesda > 5% malignancy group were: solid or almost completely solid composition (n=53, 62.3%), very hypoechoic echogenicity (n=3, 75%), wider-than-tall shape (n=50, 50.5%), lobulated or irregular margin (n=23, 65.7%), punctate echogenic foci (n=18, 72%), and thyroid isthmus location (n=6, 75%). Statistically significant individual ultrasonographic characteristics indicative of malignancy included solid or almost completely solid (p = 0.005), very hypoechoic echogenicity (p = 0.046), margin lobulated or irregular (p = 0.031), and punctate echogenic foci (p = 0.015). No significant association was found in the taller-than-wide shape for differentiating malignant from benign lesions (p = 0.969).

Conclusions

Specific ultrasound characteristics identified in the ACR TI-RADS system demonstrate a stronger correlation with an increased risk of malignancy when compared with cytologic evaluation results. These characteristics include a solid composition, lobulated or irregular margins, punctate echogenic foci, and very hypoechoic echogenicity. Our findings revealed that the scale points for the taller-than-wide characteristic do not adequately represent its true influence on the risk of malignancy.

## Introduction

Thyroid nodule evaluation employs a multidisciplinary approach involving clinical analysis, laboratory tests, ultrasound findings, fine needle aspiration biopsy (FNAB), and, in specific cases, thyroid scintigraphy. High-resolution ultrasonography is the best diagnostic modality for thyroid nodule detection. It is also the standard for evaluating cervical nodal metastases and the method of choice for getting the most accurate information while performing thyroid biopsies [1].

The prevalence of thyroid nodules is high, and it varies by the method that is used to identify these lesions; this includes palpation (2-7%), ultrasonography (20-76%), and autopsies of healthy individuals (50-76%) [2,3]. Despite its high prevalence, these lesions' overall risk of malignancy (ROM) is estimated to be 4-5% [4]. Incidence of thyroid nodules is on the rise; each year, an estimated 586,000 new thyroid cancer diagnoses are made worldwide, accounting for a total of 44,000 fatalities over the same period [5]. 

Overdiagnosis has been primarily cited for the rise in the incidence of thyroid nodule diagnosis. However, in recent years, the community's attention has been drawn to an increase in the diagnosis of advanced disease cases and an increase in disease-specific mortality for patients with small tumors; for this, more aggressive biologic profiles or possible unrecognized risk factors have been suggested [6]. However, there are no associations in the literature with a higher overall mortality rate [7,8].

Overdiagnosis of thyroid nodules has increased the use of unnecessary FNAB and surgical interventions, exposing patients with a favorable prognosis to additional procedural complications [8]. Due to this premise, adjustments to clinical practice guidelines have been made, leading to more recent studies suggesting a decrease in the incidence rates in countries such as the United States and the Republic of Korea [2,9]. 

The American College of Radiology (ACR) developed the Thyroid Imaging Reporting and Data System (TI-RADS) to provide practitioners with standardized, evidence-based recommendations for the management of thyroid nodules based on defined sonographic features. ACR TI-RADS's six categories include composition, echogenicity, shape, margins, and echogenic foci [10]. For nodule evaluation, radiologists choose one feature from each of the first four categories and all of the features that apply from the last category; characteristics that generate greater suspicion of malignancy are assigned a higher score. The sum of each category score determines the ACR TI-RADS level of the nodule, which varies from TR1 (benign) to TR5 (strong suspicion of malignancy), each one with a specific ROM [11]. Finally, the recommendation to perform FNAB is based on size and the category previously assigned. The system also considers follow-up recommendations to reduce unidentified malignant nodules that progress through time [12].

ACR TI-RADS is one of the most broadly used ultrasound stratification systems. Other relevant models include the ones developed by the American Thyroid Association (ATA) and the European Thyroid Association (EU-TIRADS). Even though diagnostic performance for malignancy detection of these systems has comparable results, for their respective categories 4 and 5, it has been reported in the literature that ACR TI-RADS has an overall higher specificity and the lowest rate of unnecessary FNAB realization. On the other hand, EU-TIRADS has a higher sensitivity [7,13,14].

Since its creation in 2010, The Bethesda System for Reporting Thyroid Cytopathology (TBSRTC) has become the main reporting scheme for cytology FNAB for thyroid nodule diagnosis. The system includes six possible diagnostic categories (I. Nondiagnostic, II. Benign, III. Atypia of undetermined significance, IV. Follicular neoplasm, V. Suspicious for malignancy, and VI. Malignant) each one with a specific ROM. These categories are consistent with the steps to follow in managing thyroid nodules, which can include observation, repeat FNAB, and surgical management [15,16]. 

During the last decade, molecular markers have rapidly gained relevance in the approach of thyroid nodules, specifically for the evaluation of indeterminate TBSRTC diagnostic categories (TBSRTC III and IV) these tests now play a well-established role in excluding malignancy. These risk categories account for about 25% of the total FNAB results; conversely, their ROM is estimated to be 5-30%. Currently, for persisting indeterminate results after using a multimodal approach (taking into account personal patient history, ultrasonographic characteristics, cytological description, and calcitonin levels) molecular testing is an important step to consider [16,17].

Although the effectiveness of ACR TI-RADS guidelines has been made evident in the available literature, further research is needed regarding how individual ultrasonographic features correlate with cytopathological outcomes. This retrospective observational study aimed to assess the validity and impact of each ultrasonographic characteristic used in the ACR TI-RADS guidelines with respect to cytopathological evaluation by TBSRTC and to evaluate the level of correlation between each individual characteristic and its risk of malignancy.

## Materials and methods

This was a retrospective study conducted at a multicenter single-institution tertiary care center, The American British Cowdray Medical Center, Mexico City, Mexico, where all thyroid examinations were performed. Reports and medical records from 2018 to 2020 were carefully reviewed. The study was approved by the Comité de Investigación Centro Médico ABC (approval number: CMABC-24-05). All thyroid nodules were classified by the ACR TI-RADS and TBSRTC.

Study population

Patients with and without a previous history of thyroid disease were referred by their physicians for thyroid evaluation by ultrasound. A total of 117 thyroid nodules were analyzed including 89 (76.1%) female patients and 28 (23.9%) male patients with an average age of 53.9±15.3 years. Based on cytology results, nodules were categorized into two groups: those with a ROM of less than 5% (n=58, 49.6%), and those with a ROM greater than 5% (n=59, 50.4%). The group with a risk of malignancy less than 5% included TBSRTC categories I (n=8, 6.8%) and II (n=50, 42.7%), and the group with a risk of malignancy greater than 5% included TBSRTC categories III (n=9, 7.7%]), IV (n=5, 4.3%), V (n=21, 18%), and VI (n=24, 20.5%). Inclusion criteria were patients with an ultrasonographic analysis evaluated with the ACR TI-RADS and the presence of ultrasound-guided FNAB cytology results evaluated with TBSRTC. This study did not include any follow-up analysis.

Ultrasound

**Figure 1:**
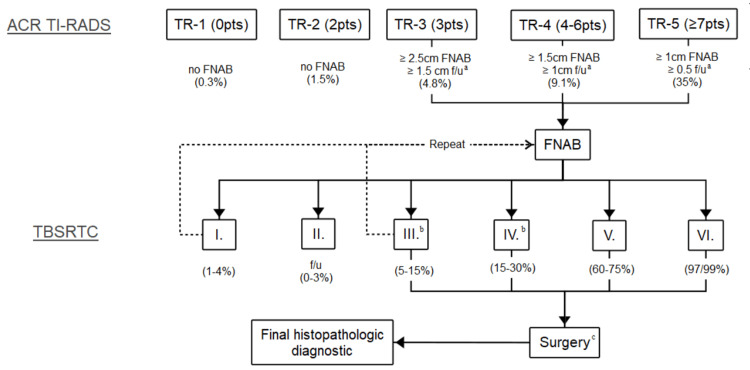
Usual thyroid nodule management pathway after ultrasound evaluation. Actual clinical decision-making depends on various other factors. ^a^ TR3 f/u one, three, and five years; TR4 f/u one, two, three, and five years; TR5 f/u annual up to years. ^b^ Bethesda III. and IV. may include molecular testing in further evaluation. ^c^ If FNAB results indicate a non-primary tumor, surgery may not be ideal. ACR: American College of Radiology; TI-RADS: Thyroid Imaging Reporting and Data System; FNAB: fine needle aspiration biopsy; TBSRTC: The Bethesda System for Reporting Thyroid Cytopathology; (%): risk of malignancy; f/u: follow-up Image Credit: Authors; References: [11,12,15,16]

All ultrasound scans were performed before FNAB, with a standardized equipment Hitachi model EUP-C532 (8-4) series KE11193302F (Hitachi Ltd, Chiyoda City, Tokyo, Japan). The scans were conducted in two medical centers within the same institution by highly experienced ultrasonography technicians. All images were reviewed by an expert radiologist, blinded to the final pathology report, and only one thyroid nodule per patient was included in the analysis. Ultrasound reports were classified according to ACR TI-RADS as described in Table 1. The usual thyroid nodule management pathway after ultrasound evaluation is illustrated in Figure 1. Imaging results were analyzed and saved using the ultrasound reports obtained from the Picture Archiving and Communication System (PACS). 

**Table 1:** Echographic characteristics and categories based on the ACR TI-RADS with scores assigned to each ultrasonographic feature ^a ^For this category, all the applicable features are summed; for the previous four, only one individual characteristic has to be assigned. ACR: American College of Radiology; TI-RADS: thyroid imaging reporting and data system Reference: [12]

Category	Individual ultrasonographic characteristic	Points
Composition	Cystic or almost completely cystic	0
	Spongiform	0
	Mixed cystic and solid	1
	Solid or almost completely solid	2
Echogenicity	Anechoic	0
	Hyperechoic or isoechoic	1
	Hypoechoic	2
	Very hypoechoic	3
Shape	Wider-than-tall	0
	Taller-than-wide	3
Margin	Smooth	0
	Ill-defined	0
	Lobulated or irregular	2
	Extrathyroidal extension	3
Echogenic Foci^a^	None or large comet-tail artifacts	0
	Macrocalcifications	1
	Peripheral (rim) calcifications	2
	Punctate echogenic foci	3

Cytology examination

Cytological reports using TBSRTC were collected from institutional medical records. These diagnoses were compared with histopathology reports made by two experienced pathologists, and then the reports were divided into benign or malignant findings.

Statistical analysis

Descriptive analysis of the prevalence and frequencies are presented for both malignancy risk groups (ROM >5%, and ROM < 5%), sex, and individual ultrasonographic characteristics. Continuous variables with normal distribution were presented with mean and standard distribution; for sensitivity and specificity calculation P < 0.05 was considered statistically significant. Contingency tables for frequency distributions between ACR TI-RADS categories were made using the Pearson chi-square test; subsequently, a bivariate logistic regression (both statistical tests that are preferred when dealing with categorical variables) was conducted to calculate the predictive capacity of each individual ultrasonographic characteristic in estimating the ROM by the category greater than 5%. P values below the 0.05 benchmark were considered statistically significant. Data was analyzed using IBM SPSS Statistics for Windows, Version 22.0 (Released 2013; IBM Corp., Armonk, New York, United States). 

## Results

Of 212 realized FNABs, 117 nodules had both ACR TI-RADS and TBSRTC reports available. Female patients were more frequently represented (n=89, 76.1%) as compared to male patients (n=28, 23.9%). In the composition category, five nodules were classified as cystic/almost completely cystic or spongiform (4.3%), 27 as mixed cystic and solid (23.1%), and 85 as solid or almost completely solid (72.6%). In the echogenicity category, three nodules were classified as anechoic (2.6%), 33 as hyperechoic or isoechoic (28.2%), 77 hypoechoic (65.8%), and four very hypoechoic (3.4%). In the shape category, 99 nodules were classified as wider-than-tall (84.6%) and 18 taller-than-wide (15.4%). In the margin category, 82 nodules were defined as smooth or ill-defined (70.1%), and 35 as lobulated or irregular (29.9%). In the echogenic foci category, 66 nodules were classified as none or large comet-tail artifacts (56.4%), 14 as macrocalcifications (12%), 12 as peripheral (rim) calcifications (10.2%), and 25 as punctate echogenic foci (21.4%). Finally, in the laterality category, 62 nodules were located at the left lobe (53%), eight at the isthmus (6.8%), and 47 at the right lobe (40.2%). Respective distributions for each group are described in Table 2. 

**Table 2:** Relationship between individual ultrasonographic characteristics and TBSRTC for risk of malignancy groups. ^*^ Pearson chi-square for ACR TI-RADS categories and TBSRTC risk of malignancy >5% TBSRTC: The Bethesda System for Reporting Thyroid Cytopathology; ACR: American College of Radiology; TI-RADS: Thyroid Imaging Reporting and Data System

Category	p^*^	Characteristics	TBSRTC <5% (N = 58), n (%)	TBSRTC >5% (N = 59), n (%)
Composition	.001	Cystic or almost completely cystic Spongiform	5 (100)	0 (0)
		Mixed cystic and solid	21 (77.8)	6 (22.2)
		Solid or almost completely solid	32 (37.7)	53 (62.3)
Echogenicity	.204	Anechoic	3 (100)	0 (0)
		Hyperechoic or isoechoic	18 (54.6)	15 (45.4)
		Hypoechoic	36 (46.8)	41 (53.2)
		Very hypoechoic	1 (25)	3 (75)
Shape	.969	Wider-than-tall	49 (49.5)	50 (50.5)
		Taller-than-wide	9 (50)	9 (50)
Margin	.031	Smooth Ill-defined	46 (56)	36 (44)
		Lobulated or irregular	12 (34.3)	23 (65.7)
Echogenic Foci	.096	None or large comet-tail artifacts	38 (57.6)	28 (42.4)
		Macrocalcifications	7 (50)	7 (50)
		Peripheral (rim) calcifications	6 (50)	6 (50)
		Punctate echogenic foci	7 (28)	18 (72)
Location	.321	Thyroid isthmus	2 (25)	6 (75)
		Right lobe	23 (48.9)	24 (51.1)
		Left lobe	33 (53.2)	29 (46.8)

**Figure 2:**
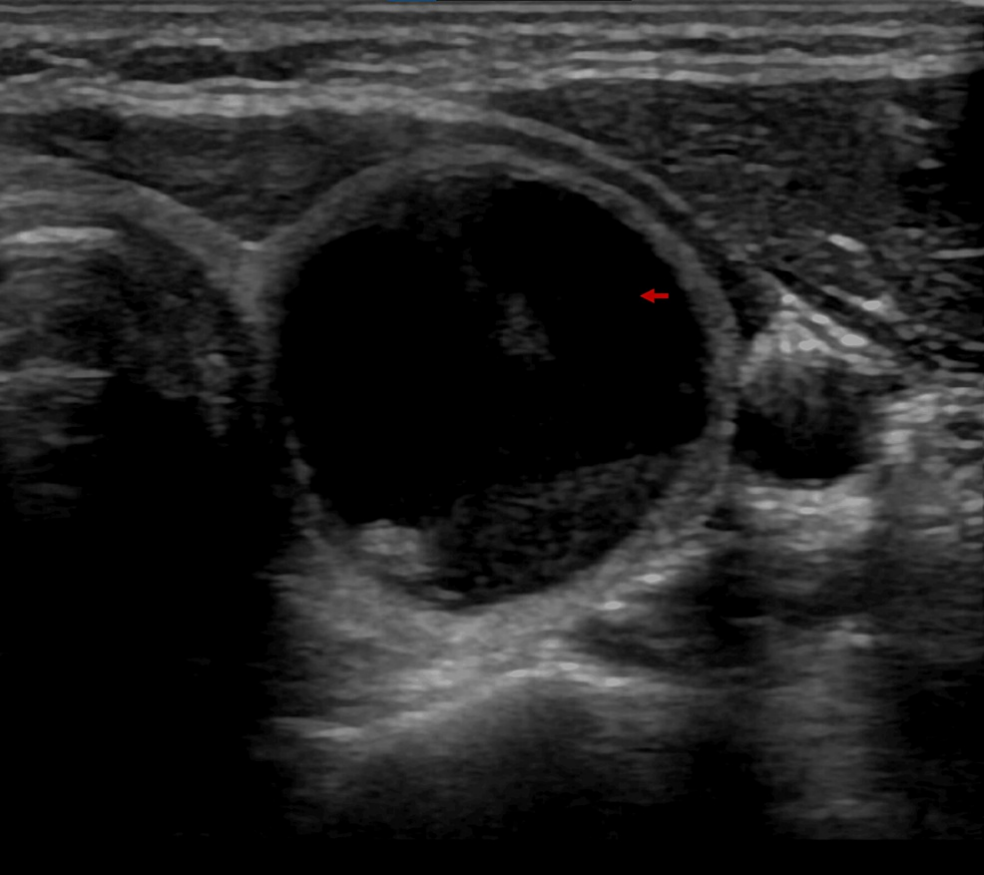
Nodular image that is predominantly cystic (red arrow), anechoic echogenicity respective to the appearance of the gland, a well-defined smooth margin, and without the presence of echogenic foci; ACR TI-RADS points score of 1. ACR: American College of Radiology; TI-RADS: Thyroid Imaging Reporting and Data System

**Figure 3:**
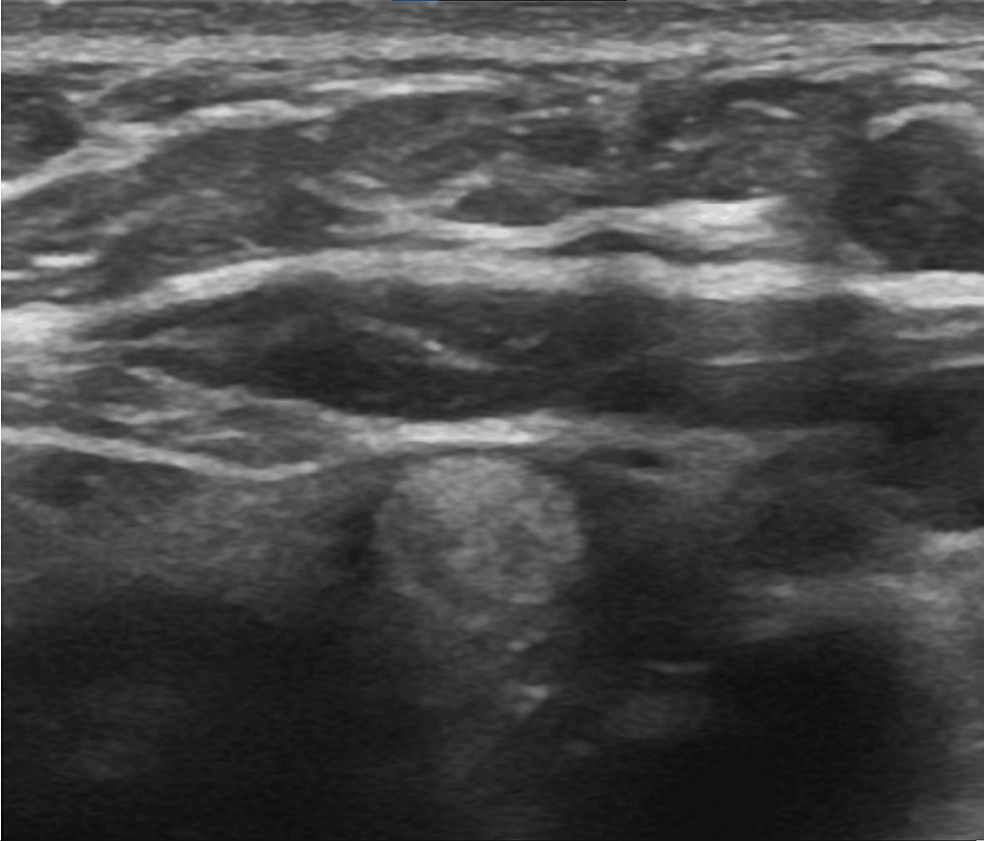
Nodular image with a solid composition, hyperechogenic echogenicity respective to the appearance of the gland and a well-defined smooth margin; ACR TI-RADS points score of 3 ACR: American College of Radiology; TI-RADS: Thyroid Imaging Reporting and Data System

**Figure 4:**
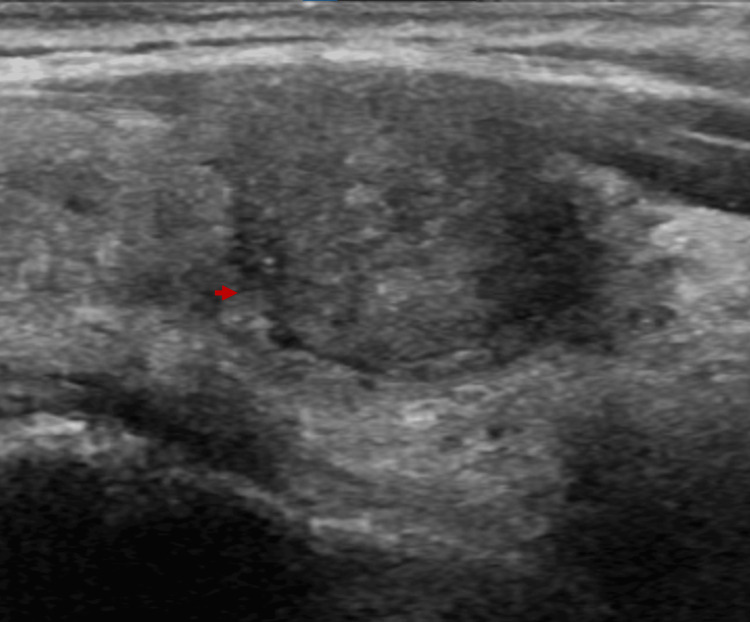
Nodular image with a solid appearance, hypoechoic echogenicity respective to the appearance to the gland, with an irregular margin (red arrow), and with some echogenic points on the periphery; ACR TI-RADS points score of 5 ACR: American College of Radiology; TI-RADS: Thyroid Imaging Reporting and Data System

**Figure 5:**
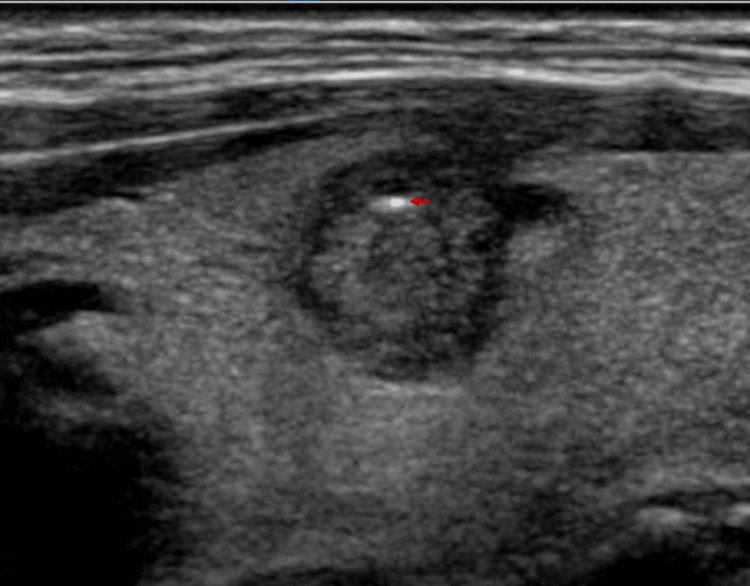
Nodular image with solid composition, hypoechoic echogenicity respective to the appearance of the gland, taller-than-wide shape, a lobulated margin, and one peripheral echogenic point (red arrow); ACR TI-RADS points score of 5 ACR: American College of Radiology; TI-RADS: Thyroid Imaging Reporting and Data System

Preliminary Pearson chi-square test testing revealed that the sample distributions closely align with the observed distributions for both composition (p < 0.001) and borders (p = 0.031) at a 95% confidence level; subsequent relevant individualized x^2^ examinations are described in the following sections. For the remaining variables (i.e., echogenicity, shape, calcifications, and laterality), the estimation of variables considering the sample did not meet the characteristics of the Pearson chi-square test (p < 0.05). The prevalence of the groups ROM < 5 % and ROM > 5 %, by each of the five main categories of ACR TI-RADS is summarized in Table 2. By our analysis, all characteristics that ACR TI-RADS assign two or three points are associated with a relatively high prevalence of malignancy > 5 % (range, 53.2%-75%), except for the taller-than-wide (50%) and peripheral (rim) calcifications (50%). Results derived from the binary logistic regression indicated that solid composition was highly significant in estimating a high ROM (p < 0.05). Likewise, cystic or spongiform compositions were significant in estimating a low ROM. Examples of characteristic ultrasonographic findings are illustrated in Figures 2-5; images were retrieved from the patient sample with respective authorizations.

## Discussion

While ultrasound plays a pivotal role in the diagnostic evaluation of thyroid nodules, the frequent occurrence of these lesions, against their low malignancy rate, represents a challenge in their diagnostic approach [2,3,18]. This study aimed to identify and analyze the individual value of each ultrasonographic characteristic used in the ACR TI-RADS scale and their ability to predict the probability of malignancy. Specifically, it focused on how each of these characteristics influences the malignancy rate and its individual impact in correlation with the TBSRTC. 

As elucidated by the female-to-male ratio of the sample (3.1), there exists a higher prevalence of thyroid nodules among women. The increased occurrence of differentiated thyroid cancers in women has been hypothesized to be the result of several factors, including differences in healthcare-seeking behaviors, sex hormones influence, and reproductive activity through life. On the other hand, the literature also describes an increased risk of poor outcomes in males, which remains inadequately understood [19]. 

Regarding the composition category, by the binary logistic regression, solid or almost completely solid feature was the only independent risk factor for predicting malignancy alone (P < 0.05) with a sensitivity of 89.8% (95%CI, 79.17%-96.18%) and a specificity of 44.8% (95%CI, 31.74%-58.46%). Observations align with findings from other studies, with an estimated ROM of 29.5-35.4% reported in the literature [20,21]. For the spongiform and cystic or almost completely cystic characteristics (P < 0.019) in correlation with the ROM < 5% group, we calculated a sensitivity of 91.4% (95%CI, 81.02%-97.14%) and a specificity of 0% (95%CI, 0.00%-6.06%), with their estimated ROM in the literature of less than 1% and 0-4.45%, respectively [22], being in correlation with our findings. 

Individual characteristics with statistically significant value included margin lobulated or irregular (p = 0.031) with a sensitivity of 39% (95%CI, 26.55%-52.56%) and a specificity of 79.3% (66.65%-88.83%), punctate echogenic foci (p = 0.015) with a sensitivity of 30.5% (95%CI, 19.19%-43.87) and a specificity of 87.9% (76.70%-95.01%), and very hypoechoic echogenicity (p = 0.046) with a sensitivity of 5% (95%CI, 1.04%-13.92%) and a specificity of 98.3% (95%CI, 90.61%-99.96%). These findings are coherent with other literature data [21-24]. 

Despite the general consensus in the literature on the utility of the shape (taller-than-wide) to differentiate malignant from benign lesions and its high degree of malignancy risk (65.3 to 86-7%) [21-23,25] for this variable, we did not find any significant association (p = 0.969). These results could be attributed to comparing two scales rather than with the final histopathologic results. Furthermore, this finding could be related to the high specificity with low sensitivity described in the literature. For better diagnostic values, adjustments to this characteristic definition have been proposed by various authors [26,27]. However, for practical purposes, the recent international consensus has not suggested changes related to this definition [22]. The additional category evaluated of laterality (right lobe, left lobe, or isthmus) added by our analysis showed no significant statistical associations.

The determination made by the ACR TI-RADS was described in three white papers. The first one recommended a standardized approach for incidental nodules detected on imaging [28]. The second one established a consensus on the terminology and criteria for ultrasound assessment, deciding the most relevant parameters by a panel of experts [10]. The third one aimed at risk stratification [12]. The system seeks to balance the benefit of identifying cancer against the costs and risks of biopsying and treating benign nodules. Using stratification scales is an appropriate method that can standardize and improve the understanding of the ultrasonographic evaluation of thyroid nodules. The use of ACR TI-RADS was chosen for our study because it is the most broadly used system with better overall performance and point-based evaluation [7,13,14]. 

Numerous authors have documented the validation and agreement between ACR TI-RADS and TBSRTC [29] and, additionally, the progressive nature of each level of the ultrasound scale [30], especially in regard to the ultrasonography-cytology correspondence where the ACR1 and ACR2 levels reach up to 90%, in association with TBSRTC category III, highlighting the "rule-out" character of the ACR TI-RADS scale [25]. These findings align with our research results.

The use of FNAB for thyroid masses, both superficial and intracavitary, dates back more than 25 years from palpation-guided puncture to US-guided aspirations of small lesions. Regarding needles, 27-gauge insulin needles were used to conventional 22-gauge hypodermic needles. In all cases, it has been confirmed that a sample of two drops of material obtained with a fine needle and using the correct technique is superior to 40 bloody smears with samples on both sides of the slides, with thick layers of fibrin, and intense artifacts due to compression and desiccation [31]. Poor material collection and spreading techniques can ruin a sample that would otherwise be sufficient and appropriate. It is essential to emphasize that the goal of the FNAB is not to puncture the nodule but to extract suitable material for diagnosis. The objective is to establish a good correspondence between clinical findings, radiology, and cytopathology. For this purpose, communication between the endocrinologist, interventional radiologist, and cytopathologist is indispensable, ideally before sample collection, to assess the immediate quality of the material and, if necessary, indicate additional passes until a sufficient and appropriate sample is obtained for direct smears and cellular block preparation.

Proceeding with the challenges inherent in the assessment of thyroid nodules, recent attention by the community has been focused on internationally accepted ultrasonographic definitions and the development of a more broadly accepted stratification system [22]. Current approaches to target areas of opportunity in the current ACR TI-RADS have included using contrast-enhanced US [32] and identifying individual ultrasonographic findings to differentiate specific subtypes of thyroid cancer [33]. Moreover, artificial intelligence is a promising tool for a new era of ultrasound diagnostics. Its future applications are extensive, targeting areas like reducing operator dependency, standardizing diagnostics, and providing assistance in areas such as analysis, segmentation, classification, and diagnosis. Advancements that are especially beneficial for less experienced practitioners or in rural areas where radiologists are limited [34].

Some limitations existed in our study. First, the analysis was conducted retrospectively, introducing a significant risk of selection bias. Second, only patients with cytology results were included; no histologic confirmation by thyroidectomy was required. Therefore, the relationship between thyroid malignancy and the diagnostic accuracy of thyroid ultrasonography tests was restricted.

## Conclusions

The use of ACR TI-RADS for the stratification of thyroid nodules effectively classifies and prevents unnecessary interventions. Other scales such as ATA and EU-TIRADS might provide complementary information, especially in cases where ACR TI-RADS results are indeterminate. Each ultrasonographic characteristic evaluated by ACR TI-RADS accurately reflects its impact on the final risk of malignancy. However, our results indicate that the points assigned by the scale for the taller-than-wide characteristic do not fully reflect its actual impact on malignancy risk. Future research should aim toward validating and integrating a multimodal approach including molecular testing, incorporating newer technologies such as artificial intelligence algorithms, and identifying ultrasonographic characteristics tailored to specific situations such as intermediate nodules, and for different types of thyroid cancer.
